# A tailored intervention does not reduce low value MRI’s and arthroscopies in degenerative knee disease when the secular time trend is taken into account: a difference-in-difference analysis

**DOI:** 10.1007/s00167-022-06949-w

**Published:** 2022-04-07

**Authors:** T. Rietbergen, P. J. Marang-van de Mheen, J. de Graaf, R. L. Diercks, R. P. A. Janssen, H. M. J. van der Linden-van der Zwaag, M. E. van den Akker-van Marle, E. W. Steyerberg, R. G. H. H. Nelissen, L. van Bodegom-Vos, for the SMART study group, P. Pander, D. J. Hofstee, R. C. I. van Geenen, K. L. M. Koenraadt, J. P. A. H. Onderwater, Y. V. Kleinlugtenbelt, T. Gosens, T. V. S. Klos, P. C. Rijk, B. Dijkstra, A. V. C. M. Zeegers, R. A. G. Hoogeslag, M. H. A. Huis in’t Veld, A. A. Polak, N. R. Paulino Pereira, T. M. J. S. Vervest, H. C. van der Veen, N. Lopuhaä

**Affiliations:** 1 Department of Biomedical Data Sciences, Section Medical Decision Making, Leiden University Medical Center, Postzone J10-s, P.O. Box 9600, 2300 RC Leiden, The Netherlands; 2 Department of Orthopaedics, University Medical Center Groningen, Groningen, The Netherlands; 3 Department of Orthopaedic Surgery and Trauma, Maxima Medical Center, Eindhoven, The Netherlands; 4 Orthopaedic Biomechanics, Department Of Biomedical Engineering, Eindhoven University of Technology, Eindhoven, The Netherlands; 5 Chair Value-Based Health Care, Fontys University of Applied Sciences, Eindhoven, The Netherlands; 6 Department of Orthopaedics, Leiden University Medical Center, Leiden, The Netherlands

**Keywords:** Choosing wisely, Degenerative knee disease, Magnetic resonance imaging, Knee arthroscopy, De-implementation, Low-value care

## Abstract

**Purpose:**

To evaluate the effectiveness of a tailored intervention to reduce low value MRIs and arthroscopies among patients ≥ 50 years with degenerative knee disease in 13 Dutch orthopaedic centers (intervention group) compared with all other Dutch orthopaedic centers (control group).

**Methods:**

All patients with degenerative knee disease ≥ 50 years admitted to Dutch orthopaedic centers from January 2016 to December 2018 were included. The tailored intervention included participation of clinical champions, education on the Dutch Choosing Wisely recommendation for MRI’s and arthroscopies in degenerative knee disease, training of orthopaedic surgeons to manage patient expectations, performance feedback, and provision of a patient brochure. A difference-in-difference analysis was used to compare the time trend before (admitted January 2016–June 2017) and after introduction of the intervention (July 2017–December 2018) between intervention and control hospitals. Primary outcome was the monthly percentage of patients receiving a MRI or knee arthroscopy, weighted by type of hospital.

**Results:**

136,446 patients were included, of whom 32,163 were treated in the intervention hospitals. The weighted percentage of patients receiving a MRI on average declined by 0.15% per month (*β* =  − 0.15, *P* < 0.001) and by 0.19% per month for arthroscopy (*β* =  − 0.19, *P* < 0.001). However, these changes over time did not differ between intervention and control hospitals, neither for MRI (β =  − 0.74, *P* = 0.228) nor arthroscopy (*β* = 0.13, *P* = 0.688).

**Conclusions:**

The extent to which patients ≥ 50 years with degenerative knee disease received a MRI or arthroscopy declined significantly over time, but could not be attributed to the tailored intervention. This secular downward time trend may reflect anoverall focus of reducing low value care in The Netherlands.

**Level of evidence:**

III.

## Introduction

Due to the ageing population, more people will suffer from degenerative knee disease in the future [23, 48]. Nowadays, around 25% of patients aged 50 years and over experience symptoms of degenerative knee disease [38, 45]. These patients suffer from complaints during walking, climbing stairs and squatting [14, 23]. Some patients also experience locking symptoms: a limited range of motion of the knee due to loose bodies or meniscal tears. Meniscal tears in this age group occur as part of a degenerative process and can be considered a feature of an early stage of osteoarthritis [17, 21].

Since 2014, clinical practice guidelines from professional orthopaedic associations [1, 4, 6, 7] as well as literature on diagnosis and treatment of these patients advise regular weight bearing radiographs including a fixed flexion view (Rosenberg view) to examine the cartilage status of the knee, and non-surgical treatment modalities including pain medication, dietary advice and exercise therapy. Routine use of an MRI for diagnosis of degenerative knee disease is not recommended for this specific patient group due to the poor association with symptoms [13, 20, 21, 32]. Similarly, arthroscopic interventions are not recommended for routine use in degenerative knee disease because limited benefits are found that are absent 1 to 2 years after surgery [16, 27, 28, 39, 40, 43]. Only when locking symptoms are present, a knee arthroscopy may be warranted. As the use of MRI and knee arthroscopy provides limited benefit, require resources and—as for any procedure—may cause harm to the patient [16, 37], both are considered low value care for patients with degenerative knee disease [29, 37].

Nevertheless, many patients are still referred for a MRI or knee arthroscopy for symptomatic degenerative knee complaints [8, 12, 15, 16, 22, 24–26, 30, 35, 42, 44]. Smith et al. [41] showed that in Australia knee MRI rates for patients aged 55 years and older increased from 216 per 100 000 to 1509 per 100 000 in 2017. Parent et al. [32] showed that only 38% of patients 50 years and over with degenerative knee disease had a plain radiograph in the 24 months preceding the MRI. Regarding knee arthroscopy, Rietbergen et al. [35] showed that in 2016 35% of knee arthroscopies in the Netherlands was performed without a documented valid surgical indication. Even more important 26% of these arthroscopies were performed on the patient’s request.

To create more awareness and reduce the routine use of MRI and knee arthroscopy in degenerative knee disease, “Choosing Wisely” recommendations were developed in several countries [2, 5, 9, 47]. These are evidence-based recommendations by professional medical specialist societies regarding use of diagnostic tests and surgical procedures. It has been shown that low value care is not reduced by a passive approach of only publishing these “Choosing Wisely” recommendations [36]. Tailored, active, interventions are more likely to succeed in orthopaedic centers that still routinely perform these low value care diagnostics and surgical procedures. The aim of this study is to evaluate the effectiveness of such a tailored intervention to reduce low value MRIs and arthroscopies in patients ≥ 50 years with degenerative knee disease in 13 Dutch orthopaedic centers (intervention group) compared with all other Dutch orthopaedic centers (control group). The hypothesis was that orthopaedic centers receiving the tailored intervention will reduce the use of low value MRI and knee arthroscopy to a greater extent than all other Dutch orthopaedic centers.

## Materials and methods

The Medical Ethical Committee (CME P16.190/NV/nv) of the Leiden University Medical Center waived the need for ethical approval for this study under Dutch law. A difference-in-difference design was used to compare the change in time trend before and after introduction of the intervention between intervention and control hospitals. Anonymized patient-level data were extracted from the Dutch National Basic Registration of Hospital Care (LBZ) [3] for all patients aged 50 years and over with knee complaints (Diagnosis Treatment Codes (DTC) 1801–1899) and a closed care trajectory in a Dutch hospital between January 1, 2016 to December 31, 2018. Dutch Hospital Data, the national organization that collects the data from all the hospitals, gave permission to use the anonymized patient data. When a patient visits a hospital the first time for knee complaints, this will generate an initial care trajectory and a follow-up care trajectory if the patient still has complaints within 120 days after the start of this initial care trajectory. All procedures including MRI and arthroscopy are assigned to this care trajectory. Patients with all their diagnostic and surgical procedures carried out in a care trajectory, were assigned to the month at which the trajectory for a specific DTC opened. All patients diagnosed and treated in intervention hospitals were included, except patients from 1 daycare orthopedic private clinic, since those patient data were not collected in the LBZ. The control group existed of patients diagnosed and treated in all other Dutch orthopaedic hospitals providing data to the LBZ in the same period (2016: *n* = 49; 2017: *n* = 55, 2018: *n* = 54).

For each anonymized patient and care trajectory, information was obtained on patient characteristics (age, sex), type of orthopaedic center (University Medical Center, Teaching Hospital, and General Hospital), Diagnosis Treatment Code (1801–1899), group (intervention or control), MRI conducted (yes/no), arthroscopy conducted (yes/no), number of MRIs conducted, number of arthroscopies conducted, month and year care trajectory opened and closed, date of MRI, date of arthroscopy, number of other care trajectories open at that time point, and type of care trajectory (initial or follow-up treatment). It was defined the period January 2016–June 2017 as before the intervention, and July 2017–December 2018 as during/after the intervention. If patients had multiple care trajectories for the same DTC (e.g. for every visit and/or treatment), it is likely that these all belong to the same care path so only the DTC for the last opened care trajectory were then used.

### Intervention

A tailored intervention was developed and implemented from July 2017 to February 2018 in the 13 intervention hospitals that participated in the ‘SMART’ (Step-down MRI’s and ARThroscopies) study. This intervention consisted of the following five components that were geared at previously identified barriers and facilitators [33] and based on previous literature were shown to have the greatest potential to reduce low value care (see also box 1):

- A local clinical leader who encouraged colleagues to follow the clinical practice guidelines (July 2017),
- Education for orthopaedic surgeons to increase their knowledge about the Dutch Choosing Wisely recommendation (July 2017),
- Training to improve their skills to manage patient expectations (September 2017),
- Feedback of performance data to orthopaedic surgeons (September 2017, February 2018), and
- A patient brochure that professionals could use in their consultations (January 2018).

### Outcomes

The primary outcome was the monthly percentage of patients receiving a MRI or knee arthroscopy in their care trajectory. Patients with degenerative knee disease were identified by diagnostic codes: arthrosis (DTC 1801) or meniscus lesion (DTC 1805). As a secondary outcome the monthly percentage of patients aged 50 years and over with a cruciate ligament injury (DTC 1820 and 1830) receiving a MRI or knee arthroscopy was calculated, which was expected not to be influenced by the tailored intervention.

### Statistical analysis

Descriptive statistics were used to compare characteristics of patients treated in intervention or control hospitals, stratified by type of hospital (general hospital, teaching hospital, university medical center) as this is known to affect the hospitals’ patient-mix.

A difference-in-difference approach was used to examine the change in monthly percentage of patients receiving a knee arthroscopy/MRI before and after introduction of the intervention between intervention and control hospitals [19]. The monthly percentage of patients receiving a MRI or knee arthroscopy was therefore also weighted for the distribution across type of hospital. The key assumption for performing a difference-in-difference analysis is a parallel trend, that is a similar time trend before introduction of the intervention for both intervention and control group [19]. This assumption was tested by visual examination and by assessing the significance of the interaction term (time (months) x group (intervention or control group)) before introduction of the intervention, which showed that the assumption was met.

For the difference-in-difference analysis, the following formula was used: weighted monthly % patients receiving a MRI or Arthroscopy = *α* + *β*1time + *β*2introduction + *β*3group + *β*4introduction × group, using linear regression analysis. In this equation, *time* covers 36 months, *introduction* refers to the period of introduction of the intervention (0 = before introduction of the tailored intervention, 1 = after introduction of the tailored intervention), and *group* indicates intervention or a control hospital (0 = control, 1 = intervention). The interaction term *introduction x group* therefore indicates whether the difference before and after introduction of the intervention differed between intervention and control hospitals. The same analyses were carried out for the secondary outcome in cruciate ligament injured patients, to assess whether there was a change in use of MRI or arthroscopy for a patient group not targeted by the intervention.

Since the components of the intervention were implemented over a period of time, these may not all have resulted in an immediate effect. Sensitivity analyses were therefore employed assuming different lag periods after the introduction of the intervention in July 2017 to account for the time it takes an intervention to affect care delivery: 3 months, 6 months, and 8 months. In addition, sensitivity analyses were performed excluding patients with a start of the initial care trajectory before January 2016, and excluding patients with a start of the initial care trajectory in November–December 2018. These analyses were done since only partial care trajectories might have been included in the data, resulting in missing MRIs or arthroscopies. All analyses were carried out with R statistics (version 3.6.2). A *p* value < 0.05 was considered significant in all analyses.

## Results

215,293 records for patients ≥ 50 years and over with degenerative knee disease were identified, which involved 136,446 patients with a care trajectory. Table 1 shows that patients did not differ in age, but that there was a difference in the distribution of sex, % DTC 1801, and % of multiple DTCs per patient between intervention and control hospitals stratified by hospital type.

**Table 1:** Background characteristics of patients with degenerative knee disease in intervention and control group at baseline

Background characteristics^a^	Intervention (*n *= 32,163)				Control (*n *= 104,283)				*P* value
	General hospital (*n *= 7691)	Teaching hospital (*n *= 23,015)	University medical center (*n *= 1457)	Total (*n *= 32,163)	General hospital (*n *= 58,724)	Teaching hospital (*n *= 43,349)	University medical center (*n *= 2210)	Total (*n *= 104,283)	
Age in years, mean (SD)	65.7 (SD 9.9)	65.8 (SD 9.7)	63.3 (SD 9.1)	65.6 (SD 9.7)	65.8 (SD 9.9)	65.7 (SD 9.9)	64.2 (SD 9.4)	65.7 (SD 9.9)	–
Male, *n* (%)^a^	3196 (9.9%)	9503 (29.5%)	642 (2.0%)	13,341 (41.5%)	23,970 (23.0%)	17,508 ( 16.8%)	903 (0.9%)	42,381 (40.6%)	< 0.001
DTC 1801, *n* (%)^a^	6159 (19.1%)	18,834 (58.6%)	1162 (3.6%)	26,155 (81.3%)	45,985 (44.1%)	36,327 (34.8%)	2064 (2.0%)	84,376 (80.9%)	< 0.001
More than 1 care trajectory open, *n* (%)^a^	3672 (11.4%)	13,144 (40.9%)	1304 (4.1%)	18,120 (56.3%)	28,432 (27.3%)	21,553 (20.7%)	838 (0.8%)	50,823 (48.7%)	< 0.001
Follow-up care trajectory, mean (SD)	1.1 (SD 1.4)	1.1 (SD 1.3)	1.5 (SD 1.6)	1.1 (SD 1.4)	1.2 (SD 1.5)	1.1 (SD 1.4)	1.3 (SD 1.6)	1.1 (SD 1.5)	–

^a^The chi-square test showed a significant difference between group and type of hospital for sex, % DTC 1801, and the percentage of patients with multiple care trajectories

**Fig. 1:**
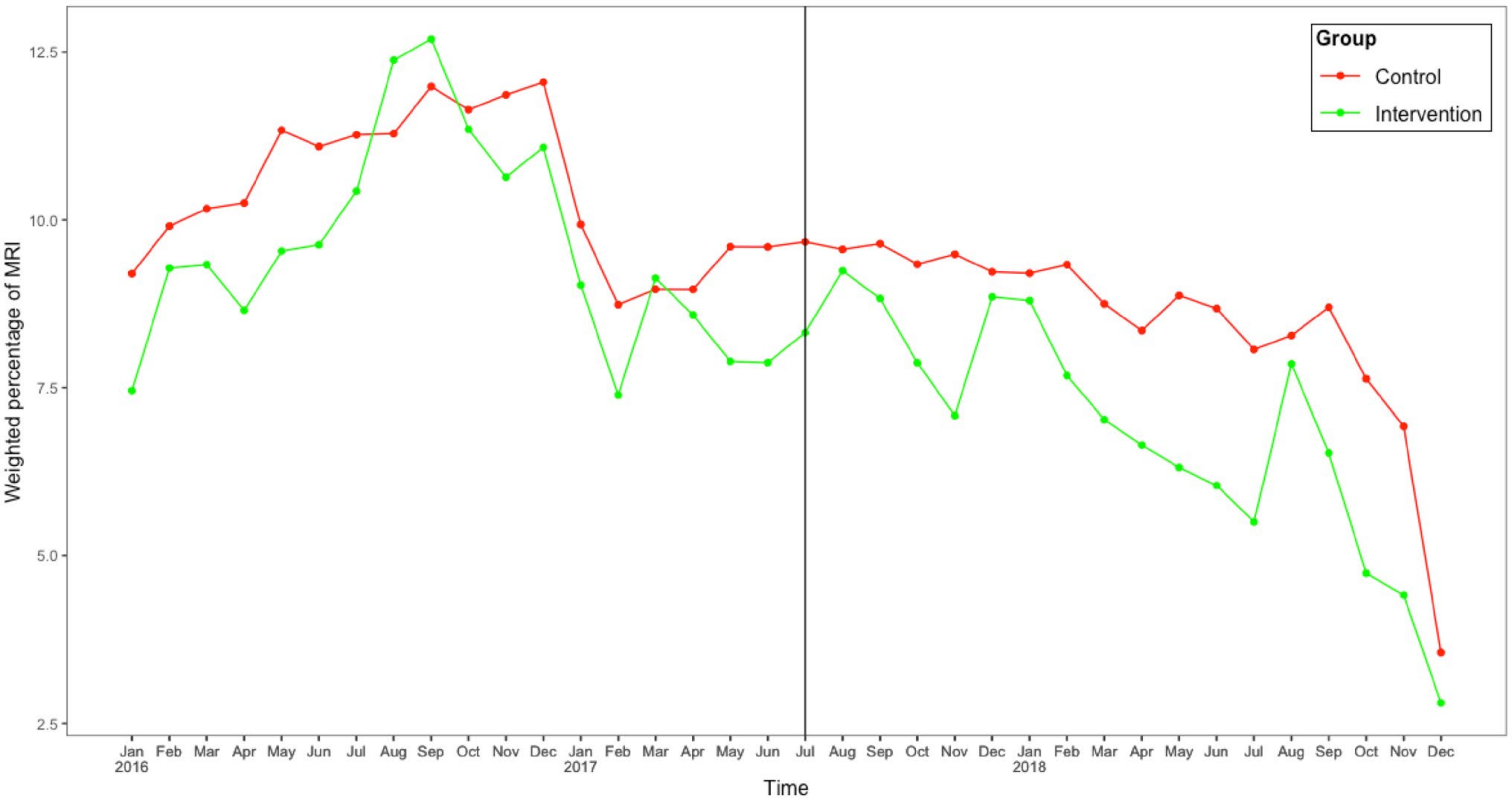
Weighted monthly percentage of patients with degenerative knee disease having a MRI

**Fig. 2:**
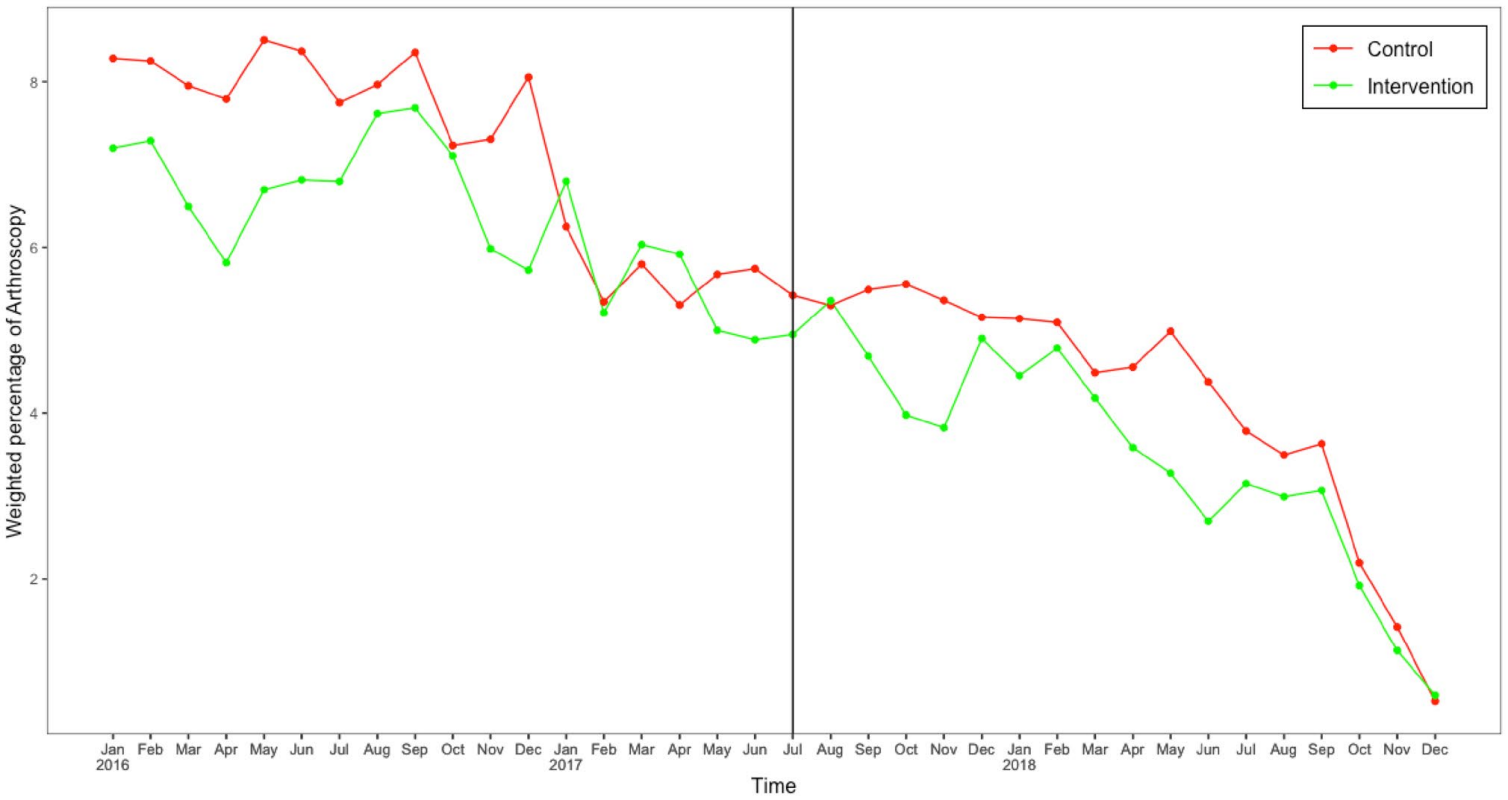
Weighted monthly percentage of patients with degenerative knee disease patients having knee arthroscopy

Figures 1 and 2 show the observed time trends in weighted monthly percentage of patients receiving MRI or arthroscopy respectively, for intervention and control hospitals with the vertical line indicating the start of the intervention. The results of the difference-in-difference analysis based on these time series data, are shown in Table 2a and b for use of MRI and arthroscopy respectively. The variable time is significant in both tables, as also apparent in the figures, indicating a secular declining trend of 0.15% per month in percentage of patients receiving a MRI and 0.19% per month for arthroscopy i.e. 5.4% and 6.8% fewer patients receiving MRI and arthroscopy during the study period. The variable group is also significant in both tables, indicating that intervention hospitals on average had lower percentages of patients receiving MRI/arthroscopy than control hospitals (0.86% lower for MRI and 0.83% lower for arthroscopy, also shown in Figs. 1 and 2 with the lines for intervention hospitals consistently lower than control hospitals. The interaction term *introduction x group* is the variable of interest to show the effect of the intervention, which is non-significant meaning that the change in percentage of patients receiving a MRI or arthroscopy before and after the introduction of the intervention, did not differ significantly between intervention and control group. In other words, the intervention did not significantly change the time trend in intervention hospitals beyond what already occurred elsewhere.

**Table 2:** Results of the difference-in-difference analyses for the weighted monthly percentage of patients receiving (a) a MRI, (b) an arthroscopy

Parameter	Estimate (SE)	*P* value
a		
Intercept	11.83 (0.41)	< 0.001
Time	− 0.15 (0.03)	< 0.001
Introduction (before vs. after)	0.72 (0.68)	0.293
Group (intervention vs. control)	− 0.86 (0.43)	0.048
Introduction × group	− 0.74 (0.60)	0.228
b		
Intercept	9.03 (0.22)	< 0.001
Time	− 0.19 (0.02)	< 0.001
Introduction (before vs. after)	0.43 (0.37)	0.246
Group (intervention vs. control)	− 0.83 (0.23)	< 0.001
Introduction × group	0.13 (0.33)	0.688

These analyses were repeated for patients with a cruciate ligament injury (diagnosis code 1820/1830) who were not targeted by the intervention (Appendix A). Again, a significant reduction in patients receiving a MRI was shown of 0.29% per month, but was non-significant for arthroscopy. As expected because the intervention was not targeted at these patients, no effect of the intervention was found as shown by the non-significant interaction term (introduction x group).

Sensitivity analyses for the different lag periods (3, 6, and 8 months) showed similar results (Appendix B and C). Other sensitivity analyses excluding patients with a DTC open before January 2016 and excluding patients with a DTC open in November 2018 or December 2018, also showed similar results (data not shown).

## Discussion

The most important finding of the present study was that a secular time trend reduced the weighted monthly percentage of patients with degenerative knee disease receiving a MRI and arthroscopy across both intervention and the control hospitals. The tailored intervention designed to reduce low value care did not further reduce the percentage of patients receiving a MRI or arthroscopy.

Previous research has shown that de-implementation of low value care in orthopaedic surgery is challenging and that providing a substitute will likely be more effective than doing nothing [46]. This might explain the lack of an effect in the present study, as no clear substitute was provided as part of the intervention, although the orthopaedic surgeons could offer patients non-surgical treatments, like advice on possible weight loss in overweight patients, physical exercises, short periods of pain killers and even adequate explanation for the presence of the knee symptoms. That substitution may be more effective is also supported by the recent study of Barlow et al. who showed that providing a substitute conservative care pathway rather than knee arthroscopy is able to reduce low value knee arthroscopies [11]. However, the study of Barlow did not use a control group, so that the observed reduction may have been part of a secular trend. Therefore, more research is needed to investigate the effectiveness of such substitute interventions.

Increasing awareness among clinicians has been shown previously to result in changes in clinical practice, particularly for issues receiving widespread attention. For instance, Kiadaliri et al. [26] showed that the development of national guideline’s recommendation against the use of knee arthroscopy in patients with knee osteoarthritis was associated with a decrease in knee arthroscopy in Sweden. In addition, Reeves et al. [33] showed, that clinical practice change occurred even before actual findings of orthopaedic trials were published. The latter phenomenon is known as the ‘rising tide’ [18] i.e. a pronounced secular trend created by social responses to a particular issue which has gained widespread attention. The current study could be another example of changing overall awareness regarding non-indicated procedures, substantiated by the growing number of studies published about the non-indicated use of MRI and arthroscopy [8, 12, 15, 16, 22, 24, 26, 30, 31, 35, 42, 44] as well as by discussions about the Dutch guideline for knee arthroscopy at meetings of the Netherlands Orthopaedic Association from 2017 onwards and the dissemination of (inter)national clinical guidelines.

Other studies have identified additional barriers that may influence decisions around performing MRI or arthroscopy, which may have been insufficiently addressed with the tailored intervention in the present study. Barlow et al. [10] found, for example, that the desire to help patients and to meet their expectations, the belief that those expectations did not involve non-surgical treatment modalities, time pressure in de clinic, and a perceived (or real) pressure from patients for an arthroscopy, were substantial barriers for reducing the use of arthroscopy in knee osteoarthritis. For the Netherlands, Rietbergen et al. have previously shown relevant barriers and facilitators for reducing the use of knee arthroscopy in degenerative knee disease which informed the intervention in the present study [34]. These barriers included orthopedic surgeons’ beliefs in the added value of MRI’s and knee arthroscopies as well as positive experiences with MRI’s and knee arthroscopies among friends and family in the patient’s environment, which both may influence the decision making for MRI and arthroscopy [34].

A strength of this study is that to a control group was included to take into account any secular time trends and separate this for the intervention effect. In the absence of such a control group, the change over time might be incorrectly attributed to the introduction of the intervention, as may have been the case in previous studies [11]. However, there are also some limitations that should be noted. First, the percentage of patients with a low value MRI or arthroscopy may have been overestimated, as in some patients there may be a valid reason for a MRI or knee arthroscopy (e.g., a truly locked knee; an extension limitation of the knee due to an intra-articular blockage) [33], which cannot be deducted from the administrative data that were used in this study. Secondly, data of orthopaedic private clinics were not available in the LBZ database so the results of this study cannot be generalized to these centers. However, a previous study [35] showed that these orthopaedic private clinics perform low value care in this patient group comparable to other hospitals. Thus it is likely that they will have been influenced by the same time trend observed in all other Dutch hospitals.

The findings of this study emphasize that it is unclear when additional quality improvement interventions are needed to reduce low value care, and when the ‘rising-tide’ phenomenon is enough to increase awareness and to implement new insights from trials or guideline recommendations. More qualitative research is needed to gain further insight into the ‘rising tide’ phenomenon, identifying when interventions are needed to de-implement low value care. Based on the findings of the study, orthopaedic surgeons are advised to improve their care by considering for which patients MRI or arthroscopy has limited value and by explaining to patients why MRI or arthroscopy has limited value, potentially supported by patient brochures.

## Conclusions

This study showed that the weighted monthly percentage of patients ≥ 50 years with degenerative knee disease who receive a MRI or arthroscopy was reduced across both intervention and control hospitals as part of a secular trend. The tailored intervention did not have an additional effect beyond this secular downward time trend.

Box 1. SMART intervention

1. Clinical champions (July 2017): local clinical leaders who encouraged colleagues to follow the clinical practice guidelines developed for diagnosing and treating patients aged 50 years and over with degenerative knee disease (e.g. during team meetings about patients).
2. Education to increase knowledge about the Dutch Choosing Wisely recommendation (July 2017): to increase knowledge of orthopaedic surgeons about the Dutch Choosing Wisely recommendation against the use of low value MRIs and knee arthroscopies for diagnosis and treatment of degenerative knee disease in patients aged 50 years and over, clinical champions were educated about this recommendations and the corresponding literature. Clinical champions subsequently disseminated this information among their colleagues using a power point presentation that was prepared by the research team.
3. Training to improve skills to manage patient expectations (September 2017)): to improve orthopaedic surgeons’ skills to manage expectations regarding the value of MRI and arthroscopy within diagnosis and treatment of degenerative knee disease, orthopaedic surgeons and residents were trained how to explain patients why it is not recommended to perform an MRI or knee arthroscopy for patients with degenerative knee disease. This was done in a meeting in each hospital/private clinic making use of videos of a consultation with a patient with degenerative knee disease. These videos included were developed in collaboration with specialised Dutch orthopaedic surgeons (RJ, RD, EvL), and included scenarios in which an orthopeadic surgeon prescribed an MRI or arthroscopy, but also scenarios in which the orthopaedic surgeons succeeded to explain to the patients that a MRI and/ or arthroscopy were not indicated. These videos were used to initiate a discussion among colleagues.
4. Feedback of data regarding MRI and arthroscopy use (September 2017, February 2018): data about the total number of patients with degenerative knee disease (including diagnosis code 1801 arthrosis and 1805 meniscus lesion), and the number of patients with degenerative knee disease who received a MRI and/ or arthroscopy was requested from each participating hospital/private clinic from 2016 until 2018. The data were analysed and presented twice to all orthopaedic surgeons in the participating hospitals/private clinics with a specialization in the treatment of knee injuries and to the residents (September 2017 and February 2018). Feedback was presented once by a researcher and once by the clinical champion or by e-mail.
5. Patient Brochure (January 2018): a patient brochure was developed to provide patients information about degenerative knee complaints, recommended treatments and an explanation why and in which cases MRI’s and arthroscopies can be regarded as low value care in diagnosis and treatment of degenerative knee disease. The patient brochure could be used during the consultation or could be given after the consultation to provide information about a stepped care strategy and the risks of an MRI and knee arthroscopy.

## Appendix

### Appendix A: difference-in-difference analysis MRI and arthroscopy for 1820/1830

Table 3.

**Table 3:** Results of the difference and difference analyses for the percentage of patients with (a) a MRI for 1820/1830, (b) an arthroscopy for 1820/1830

Parameter	Estimate (SE)	*P* value
a		
Intercept	13.36 (1.67)	< 0.001
Time	− 0.29 (0.12)	0.018
Introduction (before vs. after)	1.52 (2.76)	0.584
Group (intervention vs. control)	2.19 (1.74)	0.213
Introduction × group	− 1.04 (2.47)	0.675
b		
Intercept	25.84 (2.48)	< 0.001
Time	− 0.14 (0.18)	0.417
Introduction (before vs. after)	0.30 (4.09)	0.941
Group (intervention vs. control)	1.96 (2.58)	0.451
Introduction × group	− 0.25 (3.65)	0.945

### Appendix B: sensitivity analyses MRI for different lag periods (3, 6, and 8 months)

Table 4.

**Table 4:** Results of the difference and difference analyses for the percentage of patients with a MRI, (a) 3 month lag period, (b) 6 month lag period, (c) 8 month lag period

Parameter	Estimate (SE)	*P* value
a		
Intercept	11.70 (0.45)	< 0.001
Time	− 0.13 (0.03)	< 0.001
Introduction (before vs. after)	0.46 (0.81)	0.574
Group (intervention vs. control)	− 0.86 (0.44)	0.056
Introduction × group	− 0.89 (0.65)	0.180
b		
Intercept	11.22 (0.46)	< 0.001
Time	− 0.19 (0.02)	< 0.001
Introduction (before vs. after)	2.17 (0.62)	< 0.001
Group (intervention vs. control)	− 1.27 (0.64)	0.053
Introduction × group	0.03 (0.73)	0.967
c		
Intercept	11.23 (0.47)	< 0.001
Time	− 0.19 (0.02)	< 0.001
Introduction (before vs. after)	2.15 (0.63)	0.001
Group (intervention vs. control)	− 1.27 (0.65)	0.057
Introduction × group	0.01 (0.75)	0.989

### Appendix C: sensitivity analyses Arthroscopy for different lag periods (3, 6, and 8 months)

Table 5.

**Table 5:** a. Results of the difference and difference analyses for the percentage of patients with an arthroscopy, (a) 3 month lag period, (b) 6 month lag period, (c) 8 month lag period

Parameter	Estimate (SE)	*P* value
a		
Intercept	9.07 (0.25)	< 0.001
Time	− 0.19 (0.02)	< 0.001
Introduction (before vs. after)	0.56 (0.44)	0.211
Group (intervention vs. control)	− 0.83 (0.24)	0.001
Introduction × group	0.08 (0.36)	0.831
b		
Intercept	8.90 (0.26)	< 0.001
Time	− 0.19 (0.01)	< 0.001
Introduction (before vs. after)	0.45 (0.35)	0.209
Group (intervention vs. control)	− 1.40 (0.37)	< 0.001
Introduction × group	0.84 (0.42)	0.051
c		
Intercept	8.91 (0.26)	< 0.001
Time	− 0.20 (0.01)	< 0.001
Introduction (before vs. after)	0.43 (0.35)	0.223
Group (intervention vs. control)	− 1.40 (0.36)	< 0.001
Introduction × group	0.83 (0.42)	0.052
